# Adherence to the Mediterranean Diet in College Students: Evaluation of Psychometric Properties of the KIDMED Questionnaire

**DOI:** 10.3390/nu12123897

**Published:** 2020-12-20

**Authors:** Miguel Alejandro Atencio-Osorio, Hugo Alejandro Carrillo-Arango, María Correa-Rodríguez, Andrés Felipe Ochoa-Muñoz, Robinson Ramírez-Vélez

**Affiliations:** 1 Grupo de Investigación en Deporte de Rendimiento (GRINDER), Programa de Educación Física y Deporte, Universidad del Valle, Santiago de Cali 76001, Colombia; miguel.atencio@correounivalle.edu.co (M.A.A.-O.); hugo.carrillo@correounivalle.edu.co (H.A.C.-A.); 2 Grupo de Investigación en Actividad Física y Salud (GIAFS), Institución Universitaria Escuela Nacional del Deporte, Santiago de Cali 76001, Colombia; 3 Instituto de Investigación Biosanitaria Granada (IBIS Granada), Department of Nursing, Faculty of Health Sciences, University of Granada (UGR), 18012 Granada, Spain; macoro@ugr.es; 4 Escuela de Estadística, Facultad de Ingeniería, Universidad del Valle, Santiago de Cali 76001, Colombia; andres.ochoa@correounivalle.edu.co; 5 Navarrabiomed, Hospital Complex of Navarra (CHN)—Public University of Navarra (UPNA), Navarra Health Research Institute (IdisNa), Pamplona, C/Irunlarrea 3, 31008 Pamplona, Spain; 6 Center for Biomedical Research in Network on Healthy Fragility and Aging (CIBERfes), Carlos III Health Institute, 28029 Madrid, Spain

**Keywords:** Mediterranean diet, reliability, validity, latin-american

## Abstract

No prior studies have examined the reliability properties of the 16-item Mediterranean Diet Quality Index (KIDMED) questionnaire among young adults from a non-Mediterranean country. The objective of this study was to determine the psychometric properties in terms of the reliability and validity of the KIDMED questionnaire in young adults from Colombia. A cross-sectional validation study was conducted among 604 Colombian college students (47.51% men and 52.48% women; mean age of 21.60 ± 2.02 years). Kappa statistics were used to assess the reliability of the KIDMED questionnaire. A categorical principal components analysis was used to determine validity. Based on the KIDMED score, 58.4% of students had a good adherence to the Mediterranean diet (MedDiet). Good agreement in the general score of the questionnaire was observed (κ = 0.727, 95% confidence interval = 0.676 to 0.778, *p* < 0.001). A five-factor model was identified which explained almost 51.38% of the variability, showing the multidimensionality of the questionnaire. In conclusion, this study provides reasonable evidence for the reliability and validity of the KIDMED questionnaire for assessing adherence to MedDiet in college students within a Latin American country. The evaluation of the psychometric properties of this tool in early adulthood and in a non-Mediterranean country will be useful in clinical practice and epidemiological research, since practitioners and health researchers now have a valid and reliable short scale.

## 1. Introduction

Health behaviors characterized as lifestyle factors are the cornerstone of the prevention and management of cardiovascular diseases (CVD) and chronic diseases such as type 2 diabetes mellitus or metabolic syndrome [1]. A healthy diet is considered one of the most important factors for promoting overall health [2]. Diet has been established as a key modifiable determinant for CVD incidence and life expectancy [3]. The Mediterranean diet (MedDiet) is characterized by a high consumption of vegetables, legumes, fruits and nuts, unrefined cereals, olive oil, a moderate intake of fish, a low to moderate intake of dairy products, a low intake of meat and poultry, and a moderate alcohol intake (which comes primarily from wine); this diet is widely proven to have protective health effects against cardiovascular and metabolic diseases [4,5].

Recently, an increasing number of validated diet quality indexes have been developed to investigate diet quality among different populations [6]. In particular, different scoring methods to assess adherence to the MedDiet have been created among Mediterranean and non-Mediterranean populations [7,8]. The Mediterranean Diet Quality Index (KIDMED) questionnaire, developed by Serra-Majem et al., is one of the most internationally used measures to assess adherence to the MedDiet [9].

Early adulthood, defined as a period between 18 and 25 years of age, is a transition from adolescence to adulthood characterized by developing routines, habits, and preferences which persist throughout adulthood. During this period of life, young people experience rapid changes and build a sense of self and identity, acquiring autonomy in decision-making [10]. This stage in life has potentially relevant implications for health in later adulthood [11]. Evidence supports that interventions during this stage of life are pivotal for later health, reducing the risk of chronic diseases [12]. Young adults have often been reported to have unhealthy dietary habits including higher consumption of fast food, sugar-sweetened beverages, and alcohol [13,14,15]. They have also been shown to be the age groups most associated with a deteriorated MedDiet profile [16].

To the best of our knowledge, only one study conducted in a Mediterranean European country has determined the reliability of the KIDMED questionnaire among college students [17]. No prior studies have examined the psychometric properties of this tool in a cohort of young adults from a non-Mediterranean country such as Colombia. Taking into account that diet quality in early stages in life is strongly associated with the risk of several chronic diseases at later ages, and given the high prevalence of obesity and metabolic syndrome among Colombian university students [18,19], it is especially relevant to determine the reliability and validity of the KIDMED tool in a specific cohort of Latin American young adults. In this context, the objective of this study is to determine the psychometric properties in terms of the reliability and validity of the KIDMED questionnaire in college students from Colombia.

## 2. Materials and Methods

### 2.1. Design and Sample

A cross-sectional study was conducted among college students recruited from a public university in Cali (Colombia). This study was a secondary analysis of data from the FRICAUN study (in Spanish, ***F****actores de **RI**esgo **CA**rdiovascular en **UN**ivesitarios*), which is a non-representative survey conducted in 2020 on collegiate students from Cali, Colombia. The main objective of the FRICAUN study strategy is to use the university as a setting to promote interventions in terms of health promotion and disease prevention among public university students from Cali. All participants were socioeconomic status (SES) I–II (lower) to SES V–VI (higher; as determined by a scale by the Colombian government) in the capital of Cali, Valle del Cauca Department, in the Pacific region. This region is located at approximately 3°26′24″ N 76°31′11″ E and at an elevation of approximately 1018 m above sea level, with approximately 2980.169 inhabitants (metropolitan area). Exclusion factors included a clinical diagnosis of cardiovascular disease, diabetes mellitus 1 and 2, pregnancy, the use of alcohol or drugs, and, in general, the presence of any disease not directly associated with nutrition. A sub-sample of volunteers consisted of 604 active students (47.51% men and 52.48% women; mean age of 21.60 ± 2.17 years). A comprehensive verbal description of the nature and purpose of the study was given to the collegiate students. Written informed consent was obtained from all participants. The study was approved by the Institutional Committee for the Review of Human Ethics (CIREH) at the Universidad del Valle (ID-001-020, Internal Code 233-019 of 18 March 2020). The study was conducted according to the ethical standards established in the 1961 Declaration of Helsinki (as revised in Hong Kong in 1989 and in Edinburgh, Scotland, in 2000). All assessments were performed by trained staff.

### 2.2. Data Collection

Anthropometric measures including height and weight were assessed. Height was measured with a portable stadiometer with a precision of 0.1 mm and a range of 0–2.50 m, (Seca^®^ 274, Hamburg, Germany) and body weight (kg) was measured using an electric scale (Model Tanita BC-420^®^, Tokyo, Japan) with an accuracy of within 100 g. Body Mass Index (BMI) was calculated using the formula proposed by Quetelet, where BMI = body weight (kg)/height (m^2^). Participants were asked about sociodemographic data including SES, living area, and ethnicity.

To assess adherence to the MedDiet, the Spanish version of the KIDMED questionnaire was used [9]. This tool comprises 16 questions based on the assessment of eating habits. Each question has a “yes” or “no” answer, and responses vary between -1 (negative connotation) and +1 (positive connotation). Twelve questions are positively scored and four are negatively scored. Total KIDMED scores range from 0 to 12 and are classified as follows: ≥8 points, good (optimal MedDiet); 4–7 points, average; and ≤3 points, poor. The questionnaire was self-administered and was given at the baseline and after a one-week period. The same procedure was done on the second occasion (which took place after another one-week period to eliminate the influence of the first test responses on the results of the retest). The testing procedure was conducted from March to April 2020.

### 2.3. Statistical Analysis

The statistical packages SPSS 25.0 (IBM, Armonk, NY, USA) and JASP Statistical Software (https://jasp-stats.org/; 2018), and the macro (Lavaan SEM, LISREL) were used for the data analyses. The Kolmogorov–Smirnov statistic was applied to verify data normality. The data were expressed in the mean ± standard deviation and frequency and percentages. All numerical-type variables had normally distributed data. Independent two-tailed t-tests for continuous variables and chi-square (χ^2^) tests for categorical variables were used to examine sex differences. The appropriate sample size was at minimum 430 participants, taking into account a moderate effect size (w = 0.5), an α level of 0.05 and a statistical power of 0.95 (1 − β). Test-retest reliability was assessed by calculating the kappa coefficient and the corresponding confidence interval. The McNemar test was used to analyze the systematic differences in categorical variables.

Secondly, a categorical principal components analysis was performed to test the factor structure. We used the Kaiser–Meyer–Olkin test to rule out that the correlations between the items constitute an identity matrix, which would discourage the use of exploratory factor analyses (EFA). The degree of deviation of scores from normal distribution was analyzed by examining asymmetry and kurtosis. Because the Mardia coefficient was found to be high (189.48) for the confirmatory factor analysis (CFA), the maximum likelihood estimation method was used along with the bootstrapping procedure. A model may be considered a “good fit” if the Goodness of Fit Index (GFI), Comparative Fit Index (CFI); Tucker–Lewis Index (TLI); Bentler–Bonett Non-normed Fit Index (NNFI); Bentler–Bonett Normed Fit Index (NFI) are above 0.95 (>0.90 acceptable) and the Root Mean Square Error of Approximation (RMSEA) is below 0.05 (<0.08 acceptable), with its respective Confidence Interval at 90% (CI).

## 3. Results

The characteristics of the participants were summarized separately for males and females in Table 1. The average BMI was 22.50 (4.73) kg/m^2^ for females and 22.63 (3.23) for males. Significant differences were observed between males and females with respect to age (*p* = 0.007) and body weight (*p* < 0.001). More than half of college students reported a low SES (62.5%) and most were living in an urban area (86.9%). Based on the KIDMED score, 58.4% of the participants had a good adherence to the MedDiet. No significant differences were observed between genders for SES, living area, ethnicity and KIDMED score.

**Figure 1:**
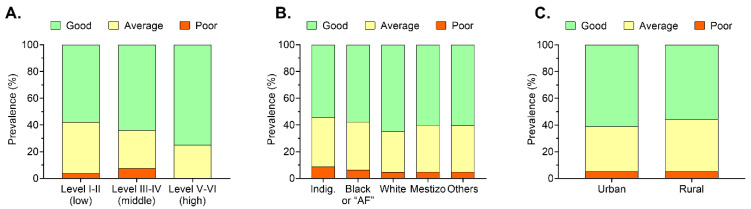
Prevalence of the Mediterranean Diet Quality Index (KIDMED) score according to the different sociodemographic factors. Data is presented in percentages (%). (**A**) Prevalence of the KIDMED score according to SES. (**B**) Prevalence of the KIDMED score according to ethnicity. (**C**) Prevalence of the KIDMED score according to living area. AF: Afrodescented.

Figure 1 shows the differences between the prevalence of the KIDMED score according to the sociodemographic factors analyzed. In relation to SES, level V–VI had higher good adherence (75.1%) than level I–I (57.9%), with statistically significant differences found. In the case of ethnicity and living area, we found a lack of statistically significant differences.

Table 2 showed basic descriptive statistics of the total sample in the KIDMED questionnaire. The highest prevalence observed was in consumption of pasta or rice almost daily (≥5 days/week) (92.1%), pulses > 1/week (89.6%), and consumption of fruit or fruit juice daily (75.6%) at the baseline. After a one-week period, similar values in most questions were reported. There were only significant changes between the first and second sample in the two questions referring to regular fish consumption (at least 2–3/week) (*p* = 0.011), and >1/week fast-food (hamburger) restaurant consumption (*p* = 0.001). Kappa statistics showed moderate to excellent agreement in each question (ranging from 0.546 to 0.883), and a good agreement in the general score of the questionnaire was found (κ = 0.727, 95%CI = 0.676 to 0.778, *p* < 0.001).

A cut-off of 0.40 was applied to simplify the principal components analysis and five factors were extracted, as shown in Table 3 and Supplementary Figure S1. Factor 1, which explained 16.29% of total variance, was characterized by consumption of fruit or fruit juice daily, second serving of fruit daily, fresh or cooked vegetables daily, and fresh or cooked vegetables > 1/day. Factor 2, which explained 11.64% of total variance, was characterized by canned commercially baked goods or pastries for breakfast, and sweets and candy several times a day. Factor 3, which explained 8.95% of total variance, was characterized by canned commercially baked goods or pastries, cereal or cereal product for breakfast, no breakfast, and dairy product for breakfast. Factor 4, which explained 7.28% of total variance, was characterized by regular fish consumption (at least 2–3/week), regular nut consumption (at least 2–3/week), and use of olive oil at home. Finally, Factor 5, which explained 7.22% of total variance, was characterized by consumption of >1/week fast-food (hamburger) restaurant and two yoghurts and/or 40 g cheese daily.

To ensure the feasibility of the EFA, the correlation matrix was evaluated (Table 4). Bartlett’s sphericity test (χ^2^ = 661.424; d.f. = 91; *p* < 0.001) affirmed that the matrix was not an identity matrix, while the Kaiser–Meyer–Olkin sampling adequacy measure (KMO = 0.670) attained a value close to 0.7. Both results indicate that it is possible to extract factors from the matrix of observed correlations.

In order to test the one-dimensionality or multidimensionality of the questionnaire, we performed categorical principal components and confirmatory analysis. A five-factor model was identified which explained almost 51.38% of the variability, showing the multidimensionality of the questionnaire (Table 4). In this model, the ratio between χ^2^ and degrees of freedom is 1.587, a value that does not exceed the limit of 3, indicating a good fit between the proposed model and the observed data. The CFI, TLI, NNFI, and NFI comfortably exceeded the 0.90 level, the value from which an acceptable fit can be considered. The SRMR value is maintained below 0.050, also indicating a better fit in the final model. Finally, an RMSEA below 0.080 is considered acceptable, whereas an RMSEA value closer to 0.050 is considered optimal.

## 4. Discussion

Since the protective effects of the MedDiet against several disorders and health outcomes have been widely demonstrated [20], there has been an interest in indexes that evaluate adherence to this dietary pattern. Here, we assessed the psychometric properties in terms of reliability and validity of the KIDMED questionnaire, which was designed to determine adherence to an optimum traditional MedDiet, in college students from Colombia. To the best of our knowledge, this is the first study to test this index in a cohort of young adults from a non-Mediterranean country. This is of special interest since the KIDMED questionnaire has been widely used in epidemiological studies and clinical contexts due to its low cost and simplicity.

Our results agree with the data reported in the review by Darmon and Drewnowski [2] that higher-quality diets are mainly consumed by better educated and/or people with high SES. Similar conclusions were reached by other investigations also suggesting that low socioeconomic groups end up having poorer diets [4]. These findings are supported, at least in part, by the fact that following a MedDiet style could represent a matter of money [4].

We observed good agreement in the general score of the KIDMED questionnaire. Furthermore, we found moderate to excellent agreement between the two samples for each question. The only significant changes found occurred in the questions referring to regular fish consumption (at least 2–3/week) and >1/week fast-food (hamburger) restaurant consumption. The wide range for each question could be explained by the fact that young adults, depending on socioeconomic status or living area, have different dietary habits. In this line, Lopez et al. [21] evaluated the relationship between food costs and adherence to food patterns, concluding that the MedDiet pattern is more expensive to follow than a western dietary pattern; this suggests that the economic barrier should be considered when counselling subjects about following a healthy diet. Additionally, Grao-Cruces [22] showed that adolescents living in rural locations had greater adherence to the Mediterranean food patterns.

In order to identify the main factors explaining the variability of the index, we carried out an EFA. A five-factor model was identified which explained almost 51.38% of the variability. This model clustered the following food consumption: consumption of fruits, vegetables, and legumes (Factor 1), consumption of pastries, bread, and sweets (Factor 2), consumption of cereals, breakfast products, and eating breakfast (Factor 3), consumption of fish, nuts, and olive oil (Factor 4), and consumption of processed foods such as hamburgers, hot dogs, pizza, yoghurts, and cheese (Factor 5). These five factors explained almost 52% of the variability. We thus showed the multidimensionality of the questionnaire.

The KIDMED instruments were applied to 604 college students from Colombia. Based on their KIDMED scores, 58.4% of participants had a good adherence to the MedDiet. Worse scores were obtained by Stefan et al. in a cohort of 276 college students from Croatia [17], and by Genc & Genc in emerging adults at a university on the western Mediterranean coast of Turkey [23]. Discrepancies can be attributed to differences in dietary habits among different countries. Examination of the nutrition patterns across the transition to adulthood, an important period that is especially salient for health behaviors, is of special interest [11]. The transition from high school to college often implies important changes to environment and resources that might result in negative health lifestyles, including poor-quality diets [15,24]. A cross-sectional study conducted in the Turkish university students showed that residency was directly related to adherence to the MedDiet, supporting the theory that students who were living with family had more positive adherence compared to the students who were living away from their family homes [23]. In the current study, almost 42% of participants had poor or average adherence to the MedDiet, suggesting a need for nutrition interventions that focus on promoting healthy dietary habits in early adulthood. Nutritional education regarding the traditional MedDiet might play a role in affecting nutrition behaviors.

This study has some limitations that should be addressed. Our study cohort comprised a well-characterized sample that only included young adults at the University of Cali (Colombia), which may limit the generalizability of the findings to other populations. Nevertheless, the sample was heterogeneous, including participants with different socioeconomic status, living in urban and rural areas, and with different ethnicities. Another limitation is the lack of data regarding the food environment for the students in Cali (whether they are residential, purchase food/meal plans, or commute and live with families). This information might be associated with the adherence to MedDiet. Unlike previous studies which only included students from one program [17], this study was carried out among students from different educational programs and disciplines. The heterogeneity of the population in the current study is a strength for validation, and suggests that KIDMED can be used in Latin American young adults with different characteristics [25,26]. Another strength of this study is the assessment of the psychometric properties of a simple questionnaire to determine adherence to the MedDiet, as other tools are limited by their complexity and length. Its validation in a specific population of Latin American young adults might facilitate its use in clinical context, since it is an easy-to-use tool to apply in primary health care.

## 5. Conclusions

For the first time, the current study provided reasonable evidence for the reliability and validity of the KIDMED questionnaire for assessing adherence to MedDiet in young adults within a Latin American country. We found good agreement in the general score of the KIDMED questionnaire. A five-factor model was identified which explained almost 51.38% of the variability, demonstrating the multidimensionality of the questionnaire. We expect that the evaluation of psychometric properties of this tool in early adulthood from a non-Mediterranean country will be useful in clinical practice and epidemiological research, since health researchers and practitioners now have a valid and reliable short scale.

## Figures and Tables

**Table 1:** General descriptive characteristics of the study participants (*n* = 604).

Characteristics	Full Sample (*n =* 604)	Female (*n =* 317)	Male (*n =* 287)	*p*-Value for Sex
Anthropometrics, mean (SD)				
Age, years	21.60 (2.17)	21.16 (2.02)	22.08 (2.57)	0.007
Body weight, kg	63.19 (12.75)	58.20 (12.45)	68.70 (10.65)	<0.001
BMI, kg/m^2^	22.56 (4.08)	22.50 (4.73)	22.63 (3.23)	0.701
Socioeconomic status, *n* (%) ^a^				
Level I–II (low)	378 (62.5)	203 (64.0)	175 (61.0)	0.206
Level III–IV (middle)	211 (34.9)	105 (33.1)	105 (36.6)	
Level V–VI (high)	16 (2.6)	9 (2.8)	7 (2.4)	
Living area, *n* (%) ^a^				
Urban	525 (86.9)	277 (87.4)	248 (86.4)	0.724
Rural	79 (13.1)	40 (12.6)	39 (13.6)	
Ethnicity, *n* (%) ^a^				
Indigenous	35 (5.8)	21 (6.6)	14 (4.9)	0.408
Black or “Afrodescented”	64 (10.6)	41 (12.9)	23 (8.0)	
White	87 (14.4)	44 (13.9)	43 (15.0)	
Mestizo	208 (34.4)	93 (29.3)	115 (40.1)	
Others	210 (34.8)	118 (37.2)	92 (32.1)	
KIDMED score (pre-test), *n* (%) ^a^				
Poor (≤3)	20 (6.3)	9 (3.1)	29 (4.8)	0.358
Average (4–7)	112 (35.3)	107 (37.3)	219 (36.3)	
Good (≥8)	185 (58.4)	171 (59.6)	356 (58.9)	

Data are reported as mean values (standard deviation, SD) or percentages ^a^.

**Table 2:** Mediterranean Diet Quality Index statistics for the total sample.

Items	Baseline		After 7 Days		McNemar Test (*p*-Value)	Kappa (κ) Statistics	95%CI Kappa
	Yes *n* (%)	No *n* (%)	Yes *n* (%)	No *n* (%)			
1. Fruit or fruit juice daily	458 (75.6)	147 (24.3)	467 (77.1)	138 (22.8)	0.335	0.683	0.614 to 0.753
2. Second serving of fruit daily	130 (21.5)	475 (78.4)	134 (22.1)	471 (77.7)	0.698	0.709	0.641 to 0.778
3. Fresh or cooked vegetables daily	434 (71.6)	171 (28.2)	420 (69.3)	185 (30.5)	0.098	0.753	0.696 to 0.811
4. Fresh or cooked vegetables > 1/day	237 (39.1)	368 (60.7)	238 (39.3)	367 (60.6)	1.000	0.546	0.478 to 0.614
5. Regular fish consumption (at least 2–3/week)	92 (15.2)	513 (84.7)	111 (18.3)	494 (81.5)	0.011	0.699	0.622 to 0.776
6. >1/week fast-food (hamburger) restaurant	401 (66.2)	204 (33.7)	369 (60.9)	236 (38.9)	0.001	0.701	0.642 to 0.760
7. Pulses > 1/week	543 (89.6)	62 (10.2)	546 (90.1)	59 (9.7)	0.775	0.550	0.438 to 0.662
8. Pasta or rice almost daily ( ≥ 5/week)	558 (92.1)	47 (7.8)	559 (92.2)	46 (7.6)	1.000	0.569	0.444 to 0.694
9. Cereal or cereal product for breakfast	405 (66.8)	200 (33.0)	395 (65.2)	210 (34.7)	0.363	0.639	0.574 to 0.704
10. Regular nut consumption (at least 2–3/week)	170 (28.1)	435 (71.8)	170 (28.1)	435 (71.8)	0.916	0.632	0.563 to 0.701
11. Use of olive oil at home	220 (36.3)	385 (63.5)	225 (37.1)	380 (62.7)	0.486	0.883	0.844 to 0.922
12. No breakfast	59 (9.7)	546 (90.1)	60 (9.9)	545 (89.9)	1.000	0.860	0.791 to 0.930
13. Dairy product for breakfast	280 (46.2)	325 (53.6)	270 (44.6)	335 (55.3)	0.282	0.767	0.715 to 0.818
14. Commercially baked goods or pastries for breakfast	65 (10.7)	540 (89.1)	55 (9.1)	550 (90.8)	0.133	0.667	0.566 to 0.768
15. Two yoghurts and/or 40 g cheese daily	81 (13.4)	524 (86.5)	82 (13.5)	523 (86.3)	1.000	0.582	0.486 to 0.678
16. Sweets and candy several times a day	224 (37.0)	381 (62.9)	223 (36.8)	382 (63.0)	1.000	0.776	0.724 to 0.829
*KIDMED index score*							
Poor (≤3)	31 (5.1)		29 (4.8)		0.568	0.727	0.676 to 0.778
Average (4–7)	208 (34.3)		219 (36.1)				
Good (≥8)	366 (60.4)		357 (58.9)				

Kappa values ≤ 0.20, 0.21 to 0.40, 0.41 to 0.60, 0.61 to 0.80, and 0.81 to 1.00 represent poor, fair, moderate, good, and excellent agreement respectively.

**Table 3:** Results from principal component analysis (PCA) and exploratory factor analysis (EFA).

Items	Factor 1	Factor 2	Factor 3	Factor 4	Factor 5
1. Fruit or fruit juice daily	0.759				
2. Second serving of fruit daily	0.691				
3. Fresh or cooked vegetables daily	0.626				
4. Fresh or cooked vegetables > 1/day	0.474				
*Explained variance (%)*	16.29				
14. Commercially baked goods or pastries for breakfast		0.723			
16. Sweets and candy several times a day		0.720			
*Explained variance (%)*		11.64			
9. Cereal or cereal product for breakfast			0.690		
12. No breakfast			0.690		
13. Dairy product for breakfast			0.656		
*Explained variance (%)*			8.95		
5. Regular fish consumption (at least 2–3/week)				0.786	
10. Regular nut consumption (at least 2–3/week)				0.488	
11. Use of olive oil at home				0.487	
*Explained variance (%)*				7.28	
6. >1/week fast-food (hamburger) restaurant					0.599
15. Two yoghurts and/or 40 g cheese daily					0.414
*Explained variance (%)*					7.22

Categorical principal components analysis varimax with Kaiser normalization.

**Table 4:** Goodness-of-fit indices for exploratory/confirmatory factor models.

Goodness-of-Fit Indices	
*Exploratory factor analysis*	
Explained variance (%)	51.38
Kaiser–Meyer–Olkin	0.670
χ^2^	661.424
d.f.	91
*p* value	<0.0001
*Confirmatory factor analysis*	
χ^2^	106.338
d.f.	67
χ^2^/d.f.	1.587
*p* value	0.002
Root Mean Square Error of Approximation (RMSEA, 90%CI)	0.051 (0.041 to 0.062)
Root Mean Square Residual (RMR)	0.047
Comparative Fit Index (CFI)	0.961
Tucker-Lewis Index (TLI)	0.946
Bentler–Bonett Non-normed Fit Index (NNFI)	0.946
Bentler–Bonett Normed Fit Index (NFI)	0.902

## Supplementary Materials

The following are available online at https://www.mdpi.com/2072-6643/12/12/3897/s1, Figure S1: PCA analysis for KIDMED questionnaire.
