# Supplementary figures and images for: Adherence to the Mediterranean Diet in College Students: Evaluation of Psychometric Properties of the KIDMED Questionnaire
Source: Nutrients. 2020 Dec 20;12(12):3897. doi: 10.3390/nu12123897 (PMC7766466; doi:10.3390/nu12123897)

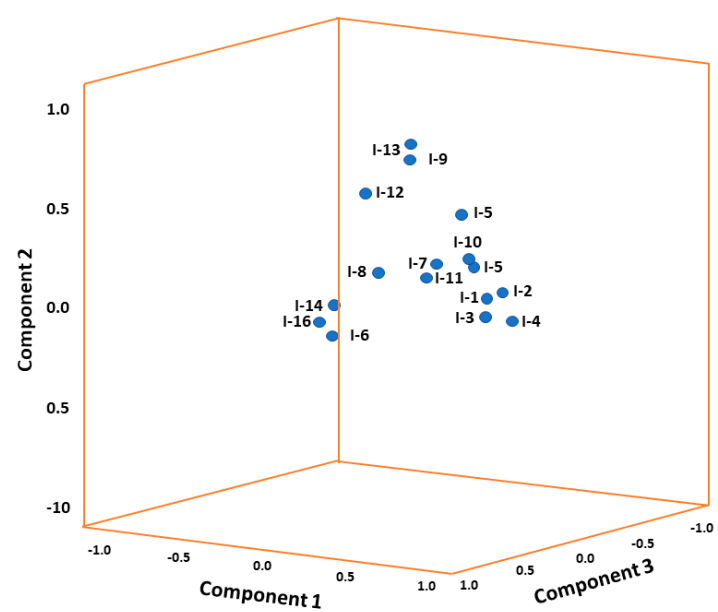

**Figure S1.** PCA analysis for KIDMED questionnaire.

Supplement: Supplementary file 1 [file nutrients-12-03897-s001.pdf]
